# PROTAC: An Effective Targeted Protein Degradation Strategy for Cancer Therapy

**DOI:** 10.3389/fphar.2021.692574

**Published:** 2021-05-07

**Authors:** Si-Min Qi, Jinyun Dong, Zhi-Yuan Xu, Xiang-Dong Cheng, Wei-Dong Zhang, Jiang-Jiang Qin

**Affiliations:** ^1^ School of Pharmaceutical Sciences, Zhejiang Chinese Medical University, Hangzhou, China; ^2^ The Cancer Hospital of the University of Chinese Academy of Sciences (Zhejiang Cancer Hospital), Institute of Basic Medicine and Cancer (IBMC), Chinese Academy of Sciences, Hangzhou, China; ^3^ School of Pharmacy, Naval Medical University, Shanghai, China

**Keywords:** PROTAC, E3 ubiquitin ligase, protein degradation, ubiquitin-proteasome system, cancer therapy

## Abstract

Proteolysis targeting chimeric (PROTAC) technology is an effective endogenous protein degradation tool developed in recent years that can ubiquitinate the target proteins through the ubiquitin-proteasome system (UPS) to achieve an effect on tumor growth. A number of literature studies on PROTAC technology have proved an insight into the feasibility of PROTAC technology to degrade target proteins. Additionally, the first oral PROTACs (ARV-110 and ARV-471) have shown encouraging results in clinical trials for prostate and breast cancer treatment, which inspires a greater enthusiasm for PROTAC research. Here we focus on the structures and mechanisms of PROTACs and describe several classes of effective PROTAC degraders based on E3 ligases.

## Introduction

As a traditional treatment method, chemotherapy plays an irreplaceable role in the cancer treatment process. The main disadvantages of traditional anticancer drugs are that most of them have poor selectivity and are easy to develop drug resistance (Mangal et al., 2017; Dong et al., 2020; Yuan et al., 2020). As a result, the targeted therapy of cancer has attracted people's attention (Zhou Y. et al., 2020; Qi et al., 2020; Yu et al., 2020). On this basis, the discoveries of new targets and small molecule inhibitors (SMIs) become powerful treatment strategies (Dong et al., 2018). In particular, the development of small molecule kinase inhibitors has become one of the most widely pursued fields in the process of drug discovery and has made great achievements in cancer treatment (Wu et al., 2015). However, after the success, the treatment strategy also faces the same problem of drug resistance as chemotherapy (Dong et al., 2020; Xu et al., 2020). Therefore, drug resistance is the main limitation for cancer therapy and needs to be solved urgently.

In recent years, a novel strategy that targets disease-related proteins for degradation has gained tremendous attention. Proteolysis targeting chimerics (PROTACs), also known as bivalent chemical protein degraders, are heterobifunctional molecules that degrade specific endogenous proteins through the E3 ubiquitin ligase pathway (Potjewyd et al., 2020). It structurally connects the protein of interest (POI)-binding ligand and the E3 ubiquitin ligase (E3) ligand through an appropriate linker (Buckley et al., 2015; Zhang et al., 2019; Kregel et al., 2020; Vollmer et al., 2020). The potential advantages of PROTAC technology may compensate for the shortcomings of traditional drug therapy, which promotes its rapid development (Toure and Crews, 2016; Sun and Rao, 2020). This paper focuses on introducing the mechanisms and the research progress of PROTAC technology, as well as summarizing the advantages of this degradation method.

## Ubiquitin-Proteasome System and Mechanism of Proteolysis Targeting Chimeric Technology

There are many approaches to protein degradation, which is very important to maintain the homeostasis of cell proteins and to regulate numerous cell processes, such as gene transcription, DNA pairing, cell cycle control, and apoptosis (Cyrus et al., 2011). Among them, the ubiquitin-proteasome system is a crucial way to specifically degrade proteins that are involved in various metabolic activities, mainly including cyclin, spindle related proteins, cell surface receptors (epidermal growth factor receptor, etc.), transcription factors (NF-κB, etc.), tumor suppressor factors such as p53, oncogene products, and intracellular denaturing proteins, whose deregulation is related to the pathogenesis of many diseases (Nam et al., 2017). UPS relies on ATP and consists of two steps: polyubiquitination of target protein and proteolysis of polyubiquitin by 26S proteolytic enzyme complex (Nandi et al., 2006).

**FIGURE 1:**
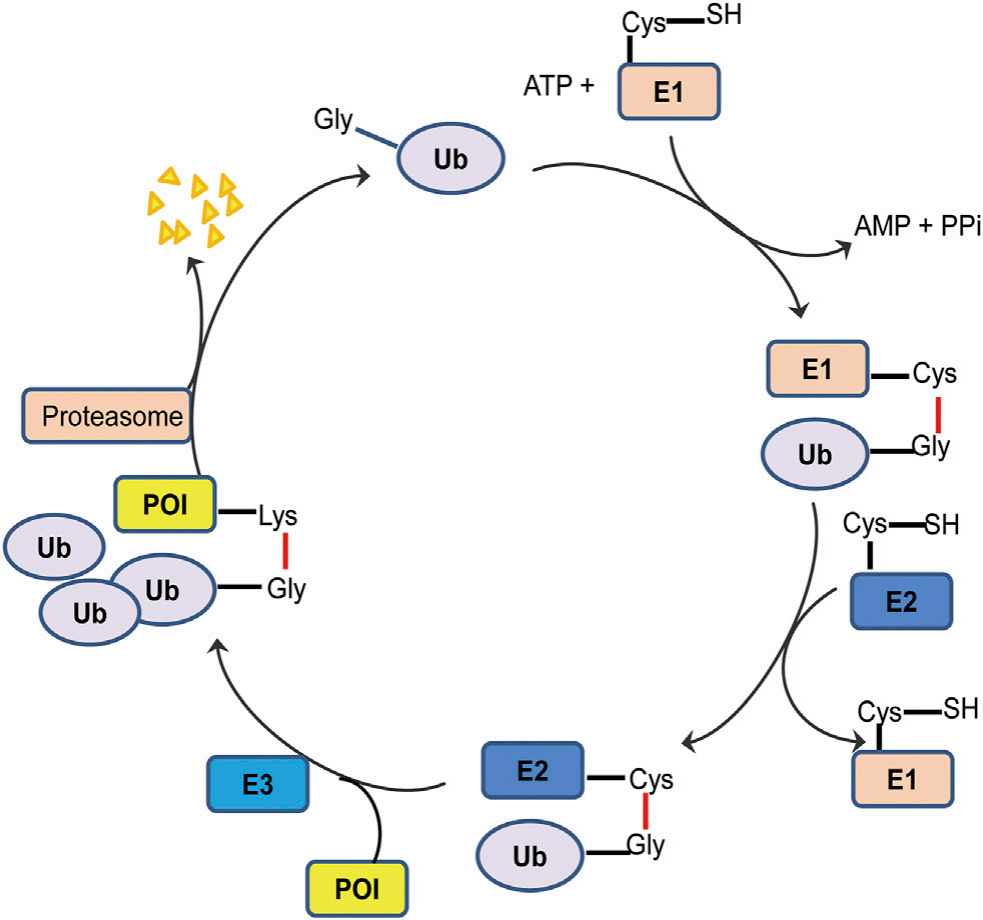
Schematic diagram of ubiquitination process. The ubiquitin tag first binds to an E1 ubiquitin activating enzyme, transfers to an E2 ubiquitin binding enzyme, and then relies on an E3 ubiquitin ligase to deliver its ubiquitin to the target protein. Ubiquitin labeled proteins are specifically recognized and degraded by proteasome.

The ubiquitin-activating enzyme E1 could form a high-energy sulfur lipid bond between the C-terminal Gly residue of the ubiquitin molecule and its own Cys residue by using ATP, and the activated ubiquitin is transferred to a ubiquitin binding enzyme E2 (Zhou L. et al., 2020). In the presence of a ubiquitin ligase E3, the ubiquitin molecule transfers from E2 to the target protein, to form an isopeptide bond with ε-NH_2_ of the Lys residue of the target protein, and then the C-terminal of the next ubiquitin molecule connects to the former at Lys48, leading to polyubiquitination (Figure 1) (Nandi et al., 2006). The ubiquitinated protein can be recognized by the cap-like regulatory particles of 26S proteasome, transported to the cylindrical core of 20S, hydrolyzed into oligopeptides by a variety of enzymes, and finally released from the proteasome to achieve the degradation of the target protein. The ubiquitin molecule, on the other hand, dissociates from the substrate and returns to the cytoplasm for reutilization (Myeku et al., 2016).

**FIGURE 2:**
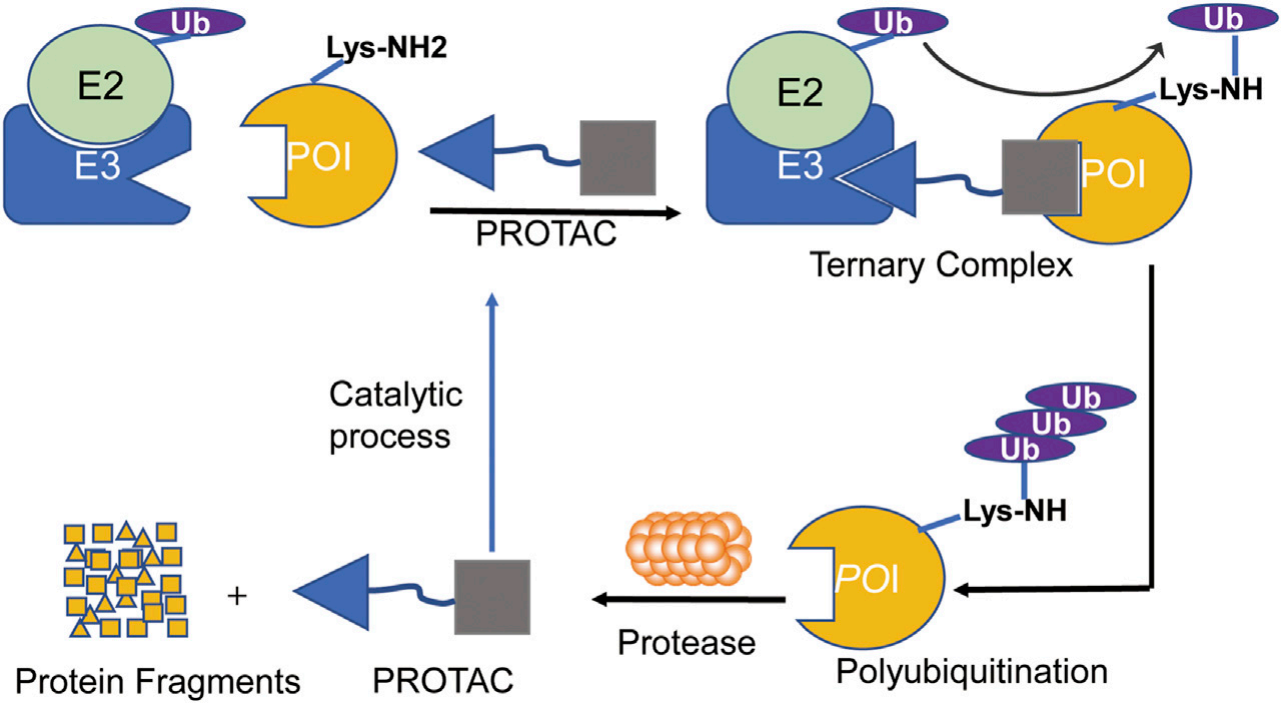
The process of PROTAC-mediated ubiquitination and proteasomal degradation of POI. PROTAC is composed of a ligand that binds to the E3 ubiquitin ligase and a ligand that binds to the target protein through a linker, which can induce the polyubiquitination and proteasome degradation of the target proteins in cells.

The mechanism of PROTACs is to use the UPS system to ubiquitinate and degrade the target protein (Wang et al., 2020b). Once the PROTAC molecules combine the target protein with E3 ligase together to form a ternary complex, which induces E3 ligase ubiquitinating the target protein to initiate the degradation process (Zou et al., 2019). The ubiquitinated target protein is recognized and degraded by 26S proteasome, which is a part of the eukaryotic cells of UPS (Figure 2). The ability of PROTACs to induce the degradation of the target protein is not limited to the binding site within the kinase domain, and it may also be achieved when the kinase activity is not the singular action of the target protein (Burslem et al., 2019).

## Comparison Among Proteolysis Targeting Chimerics, Small Molecule Inhibitorss and Monoclonal Antibodies

As two conventional treatment methods, SMIs and monoclonal antibodies (mAbs) have suffered from some inherent limitations, due to their ways of actions (Ohashi et al., 2013; Coats et al., 2019; Wolska-Washer and Robak, 2019; Lu et al., 2020). The SMIs can inhibit the biological activity of protein targets according to the action of specific active sites (Qin J. J. et al., 2017; Wang W. et al., 2019; Wang et al., 2020a). Until now, FDA has approved 62 SMIs that target about 20 different protein kinases (Roskoski, 2020). However, for most protein kinases, there is a lack of suitable active sites to target. In addition, molecule-targeted therapy is easy to induce drug resistance. All of these factors limit the development of SMIs in cancer treatment (Liu et al., 2020). mAbs are the highly uniform antibodies produced by a single B cell clone with high purity, high sensitivity, strong specificity, less cross reaction, and low cost. However, mAbs have a large molecular weight and mainly target proteins located at the plasma membrane. Besides, they need certain requirements for technology (Coats et al., 2019; Wolska-Washer and Robak, 2019).

The ligand of the target protein in PROTAC does not necessarily bind to the active site of the target protein, which overcomes the disadvantage of SMIs (Neklesa et al., 2017; Guo et al., 2019; Schapira et al., 2019). Owing to the existence of E3 ligase, PROTACs execute their functions by degrading the target proteins rather than inhibiting them, which is different from that of SMIs. Therefore, PROTAC has a great superiority in overcoming resistance caused by target mutation or overexpression when compared with SMIs. To date, PROTAC technology is applied to a variety of targets, including AR, ER, BTK, BET, and BCR-ABL to overcome resistance (Sun and Rao, 2020).

## Design and Development of Proteolysis Targeting Chimerics

The concept of PROTAC was developed by Crews and Deshaies groups in 2001, and then it has been successfully applied to multiple targets with different subcellular localization, especially in the hijacking of cancer-related kinases (Sakamoto et al., 2001; Sakamoto et al., 2003). The team first proposed a peptide-based PROTAC-1, wherein the ligand ovalbumin binds to the target protein methionine aminopeptidase-2 (MetAP-2), while the IκB, a phosphopeptide (DRHDpSGLDSM) is responsible for recruiting SCF^β−TrCP^ E3 ligase to ubiquitinate MetAP-2, leading to its degradation. In addition, the Crews and Deshaies team also verified that MetAP-2 can be degraded by Xenopus extract through the endogenous ubiquitin-proteasome pathway (Sakamoto et al., 2001). This research has opened the door of PROTAC technology, opened up a new era different from the traditional drug treatment, and paved the way for future science (Sakamoto et al., 2001).

Although there are more than 600 E3 ligases, only a few E3 ligases can be used to degrade target proteins by present PROTAC technology, including SCF^β−TrCP^, VHL (Von Hippel-Lindau), MDM2 (Murine double minute 2), IAPs (inhibitor of apoptosis proteins), and CRBN (cereblon) (Zhao et al., 2019). However, with the deepening of research, more and more E3 ligases may be developed in the future to achieve the desired degradation results. In this paper, we classify PROTACs according to E3 ligase and summarize the PROTAC degradation strategies for different target proteins (Table 1).

**TABLE 1:** Representative small-molecule PROTACs under development.

PROTAC structure	Target	E3 ligase	IC_50_ (nM)	EC_50_ (nM)	DC_50_ (nM)	References
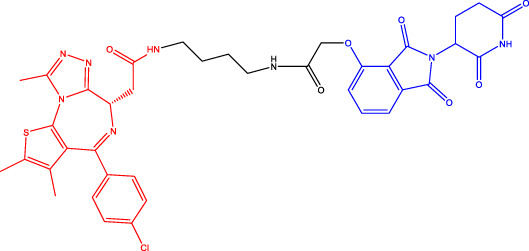 dBET1	BRD	CRBN	20	—	—	Winter et al. (2015)
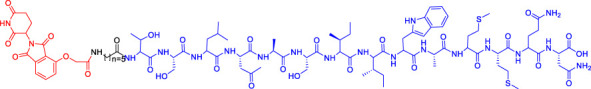 DT-6	TGF-β1	CRBN	—	—	—	Feng et al. (2020)
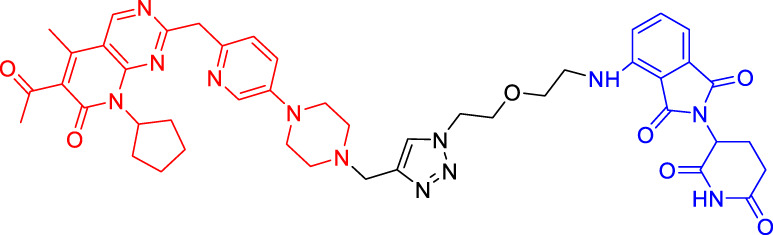 CP-10	CDK6	CRBN	—	—	2.1	Su et al. (2019)
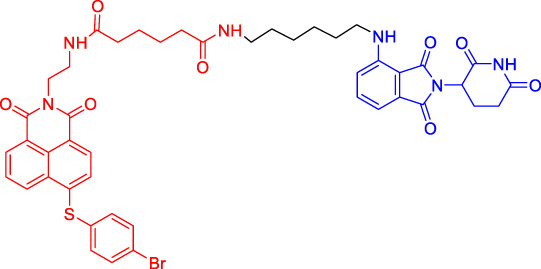 C3	Mcl-1	CRBN	—	—	700	Wang et al. (2019b)
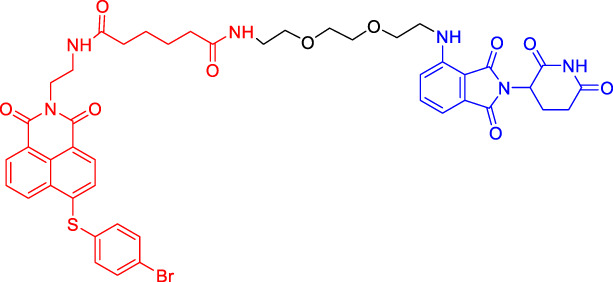 C5	Bcl-2	CRBN	—	—	3,000	Wang et al. (2019b)
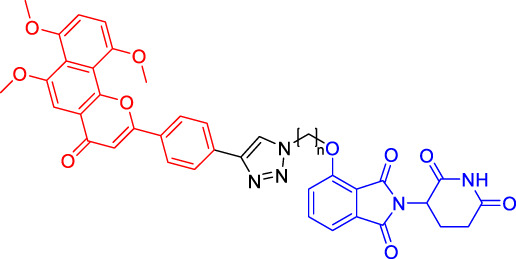 Compounds 6A-D	CYP1B1	CRBN	—	—	—	Zhou et al. (2020b)
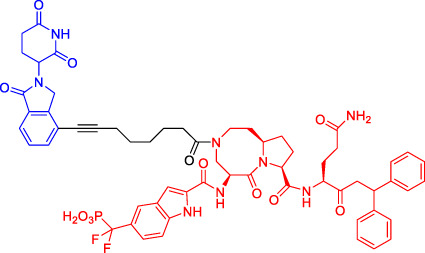 SD-36	STAT3	CRBN	13	—	60	Zhou et al. (2019)
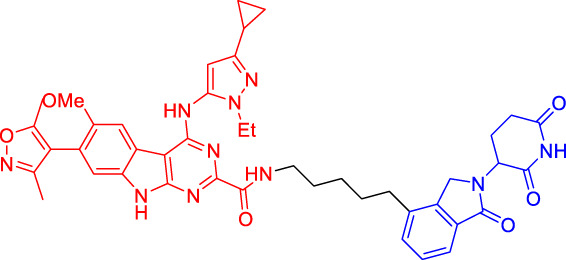 BETd-260	BET	CRBN	—	1.8	—	Shi et al. (2019)
				1.1		
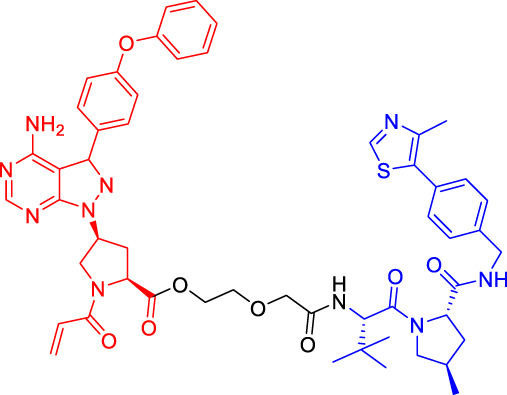 PROTAC7	BTK	VHL	—	—	136	Wang et al. (2019b)
	BLK	VHL	—	—	220	Wang et al. (2019b)
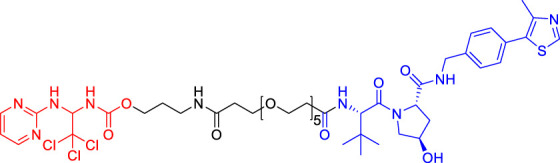 CP5V	Cdc20	VHL	2,600	—	1,600	Chi et al. (2019)
			1,990			
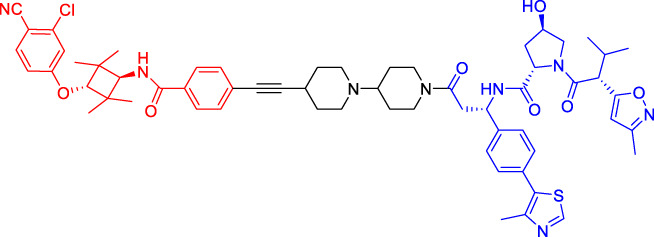 ARD-61	AR	VHL	2	—	7.2	Han et al. (2019)
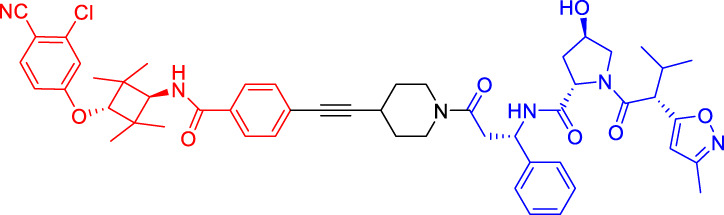 ARD-266	AR	VHL	2	—	0.5	Han et al. (2019)
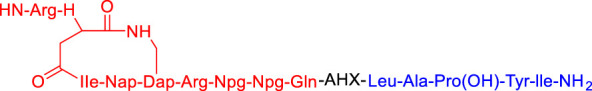 Compound I-6	ERα	VHL	9,700	—	—	Dai et al. (2020)
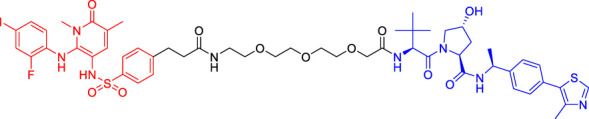 Compound 3	MEK	VHL	—	—	—	Vollmer et al. (2020)
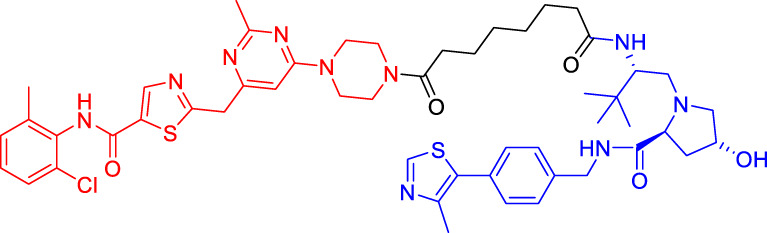 SIAIS178	BCR-ABL	VHL	24	—	8.5	Zhao et al. (2019)
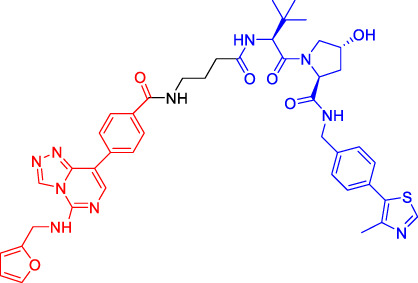 UNC6852	PRC2	VHL	247	—	—	Potjewyd et al. (2020)
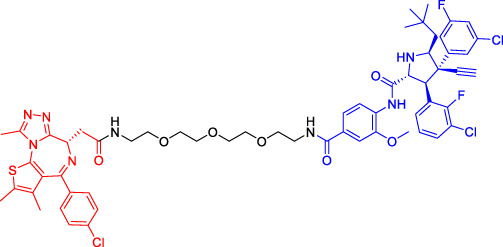 A1874	BRD4	MDM2	—	—	32	Hines et al. (2019)
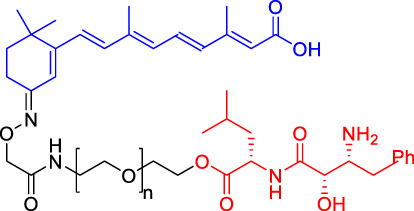 Compounds 4	CRABPs	cIAP1	—	—	—	Itoh et al. (2010)
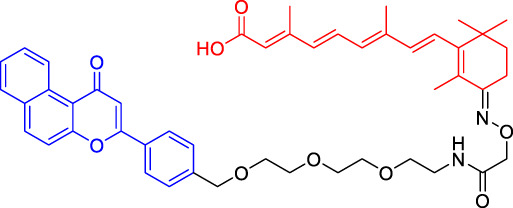 β-NF-ATRA	CRABPs	AhR	—	—	—	Ohoka et al. (2019a)
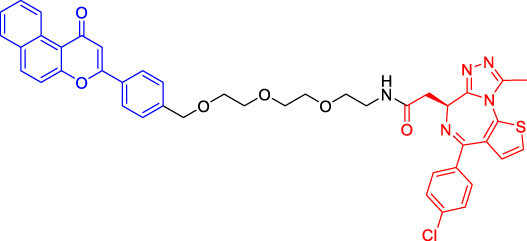 β-NF-JQ1	BRD	AhR	—	—	—	Ohoka et al. (2019a)

### Cereblon-Based Proteolysis Targeting Chimerics

CRBN, a component of a cullin-RING ubiquitin ligase (CRL) complex, is the target of thalidomide (Girardini et al., 2019). After binding to CRBN, thalidomide and its analogs inhibit the activity of CRL4^CRBN^ E3 ubiquitin ligase in human cells (Fink et al., 2018). BRD4 is a critical protein that is overexpressed in human cancer and promotes the growth and survival of cancer cells (Donati et al., 2018; Zhang F. et al., 2020). In 2015, the Bradner group has developed the first CRBN-based PROTAC, with the structure of pomalidomide capturing CRBN and BRDs inhibitor JQ1 as POI ligand. The resulting compound dBET1 has been shown to induce highly selective CRBN-dependent BET protein degradation *in vitro* and *in vivo* and delay the progression of leukemia in mice. They have demonstrated the high efficiency and specificity of dBET1 in degrading BRD family members, such as BRD2, BRD3, and BRD4, by using large-scale proteomic methods (Winter et al., 2015).

TGF-β1 is a pleiotropic cytokine and plays an important role in tumor progression (e.g., colorectal and prostate cancer). Also, it is one of the key factors of tumor cell immune escape (Sun D.-Y. et al., 2019; Dai et al., 2019). Feng’s team has developed a CRBN-based PROTAC DT-6 to degrade TGF-β1. The TGF-β1 ligand is derived from its direct inhibitor P144, and CRBN is recruited by the widely used ligand thalidomide. It has been shown that DT-6 can effectively degrade TGF-β1 in cells and reduce its secretion, which is of great significance for diseases that are correlated with the TGF-β1 signaling (Feng et al., 2020).

In light of the large effect of structure on degradation efficacy, Su’s team has designed a series of PROTACs with varying CDK6 targeting ligands, E3 ligases, and linkers. Considering that the terminal ligands of E3 ligase can also deeply affect the interaction angle between the target protein and the ligase, they have introduced flexible and rigid groups such as alkyl and alkyne into the ligand pomalidomide. To predict which ligase matches CDK6, they have also designed nutlin-3b, VH032, and Bestatin to recruit the E3 ligases MDM2, VHL, and cIAP, respectively. Three FDA-approved CDK4/6 inhibitors (palbociclib, ribociclib, and abemaciclib) have been selected as the binding ligands of the target protein CDK6, which have a strong binding ability to CDK6 with different terminal directions. Finally, it has been found that only CRBN-based PROTAC can degrade CDK6. PROTACs with shorter linkers have shown a higher capacity in CDK6 degradation, suggesting that these shorter molecules have better CRBN recruitment ability on CDK6 (Su et al., 2019).

There are many PROTACs that have been designed with pomalidomide as the CRBN ligand to degrade various POIs, such as MCL-1/BCL-2, BCL-xL, HDAC6, and BTK (Myeku et al., 2016; Sun et al., 2018; Wang X. et al., 2019; Chi et al., 2019; Yang et al., 2019; Xue et al., 2020). Protein-protein interaction (PPI) is involved in most cell processes, including cell differentiation, apoptosis, signal transduction, and transcription (Ryan and Matthews, 2005). Therefore, the role of PPI should not be underestimated, and it has been believed that the target of PPI is the next breakthrough point in disease treatment. Ye’s team has used two different BCL-2/MCL-1 inhibitors S1-6 and Nap-1 to develop two different series of PROTACs, C3 and C5 (Wang X. et al., 2019). These PROTACs have shown strong ability in PPI target degradation with DC_50_ (The 50% of maximum degradation) of 0.7 and 3.0 µM, respectively. This study has verified that PROTACs can extend the "target space" to the PPI target. It provides a selective chemical intervention for BCL-2 family protein in chemical biology research and drug discovery.

BTK, a non-receptor cytoplasmic tyrosine kinase, is involved in B cell receptor (BCR) signaling pathway and plays a key role in B cell lymphoma, so its degradation is particularly important (Hendriks et al., 2014). There are many reports on the degradation of BTK by PROTAC. Using CRBN as the E3 ligase, Crews’s team has found that MT802 can effectively degrade BTK. It has excellent degradation characteristics *in vitro* but shows a high clearance rate and short half-life *in vivo*. They have further replaced the CRBN ligand with the VHL ligand. Unfortunately, the resulting compound have shown low degradation efficiency. Finally, the structure modification of the CRBN ligand has led to the identification of SJF620, with improved druggability compared with MT802 (Jaime-Figueroa et al., 2020).

Multiple E3 ubiquitin ligases have been selected to degrade the target proteins. Ibrutinib and PLS-123, two covalent inhibitors of BTK, have been chosen as the binding part of BTK due to the high affinity and different folding structures. CRBN and VHL have been selected as the E3 ligase, which were recruited by pomalidomide and VH032, respectively. Once irreversibly combined with target kinase, an excellent degradation efficiency has been observed in living cells (Xue et al., 2020). Different from Pan’s team, CRBN and MDM2 have been selected as the E3 ligases in Rao’s study (Sun et al., 2018; Xue et al., 2020). In addition to the recruitment of CRBN by pomalidomide, RG-7112 has been designed as the ligand for MDM2 recruitment and ibrutinib and spebrutinib have been selected as the BTK ligands. It has been found that CRBN is generally more effective as E3 ligase than MDM2 (Sun et al., 2018). Besides BTK, CRBN- and VHL-PROTAC can also effectively degrade EGFR, BRD4, PLK1, and CDK2 (Zhou F. et al., 2020; Zhang H. et al., 2020; Mu et al., 2020).

In addition, Li et al. have developed a PROTAC that can degrade the cell cycle kinase Wee1 and provided a new direction for targeted cancer therapy (Jaeger and Winter, 2020; Li et al., 2020). Winzker et al. have described that PDEδ-based PROTACs can effectively and selectively reduce the level of phosphodiesterase-δ (PDEδ) in cells (Winzker et al., 2020). At the same time, it has also increased the expression of various lipid-related enzymes and the level of cholesterol precursor. The results have also shown that PDEδ plays a role in the regulation of sterol synthesis (Winzker et al., 2020). Signal transducer and activator of transcription 3 (STAT3) activation is beneficial to the survival, reproduction, metastasis, and immune escape of tumor cells (Furtek et al., 2016). STAT3 is closely related to the adverse prognosis of human cancer and has become a promising therapeutic target for cancer and other diseases. Zhou et al. have developed SD-36 as a highly selective and potent PROTAC degrader of STAT3. SD-36 can inhibit the growth of leukemia and lymphoma cell lines with highly phosphorylated STAT3 at low nanomolar concentrations *in vitro*. SD-36 can also completely and persistently regress the tumor growth in mice bearing the Molm-16 xenografts. SD-36 has been found to rapidly induce the degradation of STAT3 but has no significant effect on other STAT isoforms (Zhou et al., 2019).

Bromodomain and Extra-Terminal domain (BET) family proteins are epigenetic regulatory factors related to the expression of multiple oncogenes (Stathis and Bertoni, 2018). BETd-260 is an effective PROTAC degradation agent synthesized on the basis of BET SMIs. The *in vivo* and *in vitro* experiments have shown that it can induce a large amount of apoptosis in osteosarcoma (OS) cells and OS xenograft tumor tissues and ultimately lead to the depth and sustained inhibition of tumor growth in both mouse OS cell line-derived xenograft and patient-derived xenograft (PDX) models (Shi et al., 2019).

### Von Hippel-Lindau-Based Proteolysis Targeting Chimerics

VHL, an important tumor suppressor of clear cell renal cell carcinoma (ccRCC), is a part of the E3 ubiquitin ligase complex (Zhang et al., 2018). Its regulatory pathway involves the activity of E3 ligase, which can target hypoxia inducible factors α (including HIF1α and HIF2α) for proteasomal degradation (Pezzuto and Carico, 2018). Recent studies have shown that VHL possesses additional HIF-independent functions. For example, in VHL-deficient ccRCC, the assembly of VHL-mediated intercellular junctions is achieved through HIF-independent mechanisms (Calzada et al., 2006; Zhang and Zhang, 2018). Accordingly, there are many PROTACs that use VHL as the E3 ubiquitin ligase to degrade the target protein.

Kim’s team has also recruited CRBN and VHL by using pomalidomide and VH032, respectively (Kim et al., 2019). They are dedicated to knowing whether the E3 ligase itself can be ubiquitinated and degraded by another E3 ligase when two different E3 ligases are put together. Therefore, they have designed PROTACs to target CRBN or VHL itself. However, in all cases, the results have shown that the level of CRBN is decreased while the level of VHL is unchanged or increased, indicating that RPOTAC can ubiquitously degrade CRBN itself (Kim et al., 2019).

Chronic myeloid leukemia (CML) is a kind of malignant tumor that affects blood and bone marrow. It is characterized by the production of a large number of immature leukocytes to inhibit the normal hematopoiesis of bone marrow. BCR-ABL1 is a critical kinase in CML, which drives the over production and expansion of white blood cells in bone marrow and finally squeezes out normal cells in the bone marrow (Burslem et al., 2019). Crews lab has developed a series of PROTACs for BCR-ABL1 protein. They have used their previously developed E3 ligase VHL ligand to degrade the fusion protein (Buckley et al., 2012a; Buckley et al., 2012b). Their research further proves the great ability of the PROTAC technique, for it is not only a potential therapeutic method but also a tool to explore basic biology (Burslem et al., 2019).

Mitosis is the primary mechanism of cell proliferation, and thus inhibition of cancer proliferation can be achieved by blocking the process of mitosis. Cell division cycle 20 (Cdc20) is a key factor in mitosis, and targeting Cdc20 has been considered as a novel cancer therapeutic strategy (Wang et al., 2015). A PROTAC molecule named CP5V has been designed to induce the degradation of Cdc20, with PEG5 being used to connect the Cdc20 ligand and the VHL ligand. CP5V can effectively degrade Cdc20 and eventually overcome cell division slippage, which is the main reason for drug resistance of taxane in breast cancer treatment (Chi et al., 2019).

Overexpression of anti-apoptotic proteins such as BCL-2 and BCL-XL will promote the development and progression of cancer (Singh et al., 2019). Many SMIs targeting the BCL-2 family have been developed, such as ABT263 (a BCL-2 and BCL-xL dual inhibitor) and ABT199 (a BCL-2 selective inhibitor) (Chang et al., 2016; Naqvi et al., 2017). However, ABT263 has the obvious disadvantages of on-target toxicity and dose-limiting thrombocytopenia, which greatly limits its clinical application. Although ABT199 has become the only BCL-2 family anticancer drug approved by the FDA, it cannot be used in the treatment of solid tumors because most solid tumor cells do not rely on BCL-2 expression. The shortcomings of traditional inhibitors prompted Khan’s team to develop a PROTAC, DT2216, which can target BCL-xL protein degradation by the VHL E3 ligase (Khan et al., 2019). Compared with ABT263 (BCL-xL inhibitor), DT2216 not only has strong inhibitory effects on all kinds of BCL-xL -dependent leukemia and cancer cells *in vitro* but also has much less toxicity to platelets because of the poor expression of VHL in platelets. DT2216, either as a single drug or combined with other chemotherapeutic drugs, can effectively inhibit the tumor growth in several xenograft mouse models without causing significant thrombocytopenia *in vivo*. Their research has shown that DT2216 may have a great potential to replace the traditional SMIs as a safe and effective anticancer drug targeting the BCL-2 family (Khan et al., 2019).

Androgen receptor (AR) antagonists play a pivotal role in the treatment of metastatic castration-resistant prostate cancer (mCRPC), but they still face the problem of drug resistance. Using PROTAC technology, Han et al. have developed several AR degraders by using four different AR antagonists as the AR ligands, of which ARD-61 with ARI-16 as the AR ligand is the most effective one (Han et al., 2019). Compared with AR antagonists, ARD-61 has a better inhibitory effect on cancer cell proliferation and can overcome drug resistance, suggesting that PROTAC-mediated degradation of AR has great clinical potential. In addition, the team has also proved that even if the E3 ligands have a micromolar binding affinity to ubiquitin ligase E3, the obtained PROTAC products can still effectively degrade the target protein, which contributes to overwhelming the difficulty of seeking high active ligands for E3 ligands complex (Han et al., 2019).

It has been reported that polycomb repressive complex 2 (PRC2) is both a carcinogenic gene and a tumor suppressor gene (Gan et al., 2018). The catalytic activity of PRC2 depends on the embryonic ectodermal development (EED), enhancer of zeste homolog (EZH1) or EZH2, and suppressor of zeste homolog 12 (SUZ12) (Margueron and Reinberg, 2011). PRC2 is located at histone 3 lysine 27 (H3K27), and H3K27 trimethylation (H3K27me3) is the key mechanism for transcriptional repression (Ferrari et al., 2014). EZH2 is up-regulated in a variety of cancer types, such as breast, colorectal, and prostate cancer. The overexpression of EZH2 and the increase in the H3K27me3 level contribute to cancer cell proliferation and chemotherapy resistance, leading to a low survival rate in clinical practice. EED, EZH2, and SUZ12 are also susceptible to cancer mutations. Therefore, targeting EED and EZH2 can effectively block the catalytic activity of PRC2. UNC6852 is a PROTAC designed using the EED ligand EED226. It has been shown that UNC6852 has time- and concentration-dependent inhibitory effects on EED, EZH2, and SUZ12 of PRC2 in HeLa cells in a VHL-dependent manner, with a reduced level of H3K27me3 (Potjewyd et al., 2020).

### Murine Double Minute 22-Based Proteolysis Targeting Chimerics

P53 is an indispensable tumor suppressor that regulates cell cycle, apoptosis, DNA damage repair, and other processes (Qin J.-J. et al., 2017; Qin et al., 2018). MDM2 is one of the main inhibitors of p53; it can bind to p53 through its N-terminal domain (region I) to form the MDM2-p53 complex and reduce the activity and level of p53 (Hou et al., 2019; Wang et al., 2020a). MDM2 gene exists in the cell genome of human malignant tumors such as lung and colon cancer (Mendoza et al., 2014). Overexpressed MDM2 can be detected in many malignant tumors, so MDM2 has become an effective target for the development of anticancer drugs (Nag et al., 2013). With the emergence of PROTAC, MDM2 has also been developed as an E3 ligase to degrade AR (Sun X. et al., 2019) and BRD4 (Groppe, 2019). Nutlin-3a and Idasanutlin are usually selected as the E3 ligase ligands. Although nutlin-3a specifically binds to MDM2 with a high binding affinity, there are few PROTACs that are designed and developed based on nutlin-3a. A1874, a BRD4 PROTAC based on nutlin-3a, degraded 98% of its target protein at nanomolar concentrations and activated the p53 signaling pathway. This study has also shown that for the same target protein (e.g., BRD4), MDM2-based PROTAC has a better degrading effect than CRBN-based PROTAC (provided that in the context of wild-type p53) (Hines et al., 2019).

### Inhibitor of Apoptosis Proteins-Based Proteolysis Targeting Chimerics

Available data have confirmed that IAPs are involved in cancer and other human diseases and have been considered as a potential target for cancer treatment (LaCasse et al., 2008). Mammalian IAP protein family includes at least 8 members, among which cIAP1 and cIAP2 function as E3 ubiquitin ligases to mediate the ubiquitination of target proteins (Fulda, 2017). In 2010, Hashimoto’s team has hijacked cIAP1-E3 ligase using bestatin-methyl ester MeBS and used all-trans retinoic acid (ATRA) as a warhead to develop the first cIAP1-based PROTAC (compounds 4) to degrade cellular retinoic acid binding protein (CRABP-I/-II) (Itoh et al., 2010). Compounds 4 has been shown to induce the selective loss of CRABP-I and -II proteins in cells in a concentration-dependent manner. With the deepening of research, more and more IAP1-based PROTACs have been developed. Interestingly, unlike other PROTACs, IAP-based PROTACs have dual functions of degradation of POI and IAP, which is beneficial to the anti-tumor function and also suggests that it should be careful in design to avoid unexpected side effects (Ohoka et al., 2019b; Liu et al., 2020).

## Clinical Research on Proteolysis Targeting Chimerics

Currently, several PROTACs have entered clinical trials (Table 2), and some of them have shown encouraging results, such as ARV-110 and ARV-471. ARV-110, an oral protein degradation agent, binds AR specifically and mediates its degradation (Neklesa et al., 2018). ARV-110 completely degraded AR (DC_50_ < 1 nM) in all tested cell lines (Neklesa et al., 2019) and Oral ARV-110 (10 mg/kg) significantly inhibited the growth of enzalutamide-insensitive tumors in the PDX model (Wang et al., 2020b). ARV-110 degrades clinically relevant mutant AR proteins and retains activity in a hyperandrogen environment. The early reported data (by January 2020) from the first-in-human, phase I study of ARV-110 demonstrated its safety and tolerability in patients with metastatic castrate-resistant prostate cancer (mCRPC) (Petrylak et al., 2020). ARV-110 was administered to 18 patients at four doses, including 35 mg (*N* = 3), 70 mg (*N* = 4), 140 mg (*N* = 8) and 280 mg (*N* = 3). Among them, 12 patients received ARV-110 combined with enzalutamide (ENZ)/abiraterone (ABI), and 14 patients received prior chemotherapy. One patient administered ARV-110 280 mg experienced Grade (GR) 4 elevated AST/ALT followed by an acute renal failure while taking together with rosuvastatin (ROS). Similarly, another patient developed GR3 AST/ALT while taking ROS. Their plasma concentrations of ROS were increased accompanied by AST/ALT elevations, suggesting that concurrent ROS could produce toxic side effects. For other patients, no related GR 3/4 adverse events were reported. Generally, ARV-110 possesses an acceptable safety profile. 15 of 18 patients were evaluable for prostate specific antigen (PSA) response. Of these, two patients had a PSA reduction of more than 50% (140 mg dose group), and both of them received prior therapy including ENZ and ABI, chemotherapy, bicalutamide, radium-223 and others.

**TABLE 2:** PROTACs in clinical stage.

Drug	NCT numbers	Target	Lead indication	Phase	Toxicity profile	Preliminary efficacy data
ARV-110	NCT03888612	Androgen receptor	Prostate cancer	Phase 2	ARV-110 has an acceptable safety profile; however, co-administration of rosuvastatin with ARV-110 could produce toxic side effects.	Two of 15 patients had a PSA reduction of more than 50% (140 mg dose group); two of five patients (40%) with T878 or H875 mutations in AR had PSA reductions over 50%; two of 15 patients (13%) with wild-type AR also had PSA reductions over 50%
ARV-471	NCT04072952	Oestrogen receptor	Breast cancer	Phase 2	ARV-471 is well tolerated at all tested dose levels; no treatment-related grade 3 of 4 adverse events, and DLTs were reported. The most common treatment-related grade 1–2 adverse events are nausea (24%), arthralgia (19%), fatigue (19%), and decreased appetite (14%)	One patient (totally 21 adult patients) in ARV-471 trial had a 51% reduction in target lesion size (confirmed PR), two patients had unconfirmed PRs, and one additional patient showed stable disease, with a target lesion reduction of more than 50%; five of 12 patients (42%) achieved CBR
KT-474	NCT04772885	IRAK4	Autoimmune including AD, HS and RA	Phase 1	NR	NR
NX-2127	NCT04830137	BTK	B cell malignancies	Phase 1	NR	NR

NR, not reported yet (Recruiting Status).

According to the recent interim clinical data released by Arvinas (https://ir.arvinas.com/), in the phase 1 clinical trial, ARV-110 shows promising activity in a very late-line mCRPC patients, with PSA reductions over 50% at doses greater than 280 mg. Previous studies have shown that multiple pre-treatments will lead to tumor resistance to targeted AR therapy, and improve the heterogeneity of tumor, resulting in a decreased efficacy of AR targeted therapy. Molecular biological analysis of patients treated with ARV-110 showed that 84% of patients carried non-AR gene mutations. Among the highly heterogeneous phase 1 patients, Arvinas has identified an advanced population with a molecular definition that has a particularly strong response to ARV-110. Of the five patients with T878 or H875 mutations in AR, two (40%) had a PSA reduction of more than 50%, including one with PR confirmed by RECIST and tumor size reduction of 80%. Additionally, two of 15 patients (13%) with wild-type AR also had PSA reductions over 50%. These results suggest ARV-110 has great potentials in molecularly defined population (T878/H875) and in wild-type patients.

ARV-471 is an estrogen receptor (ER) alpha PROTAC molecule that degrades ER in ER-positive breast cancer cell lines with DC_50_ around 1 nM. It can decrease the expression of classically regulated ER-target genes and suppress the growth of ER-dependent cell lines (including cell lines expressing ESR1 variants such as Y537S and D538G) via degradation of ER. Oral administration of single agent ARV-471 (3, 10, and 30 mpk/day) shows significant anti-tumor activity in estradiol-dependent MCF7 xenografts along with ER protein reductions of over 90%. Excitingly, more pronounced tumor growth inhibition is observed (131% TGI) in the MCF7 xenograft model, accompanied by significant reductions in ER protein levels when combined with a CDK4/6 inhibitor palbociclib. Furthermore, the combination of ARV-471 and CDK4/6 inhibitor palbociclib showed great superiority over the combination of fulvestrant with palbociclib in tumor regressions. Also, ARV-471 (10 mpk) completely inhibited growth and markedly reduced mutant ER protein levels in ESR1 mutant hormone-independent PDX model (Flanagan et al., 2019). These promising results translate well into clinical trials. Recently, Arvinas has also announced ARV-471 for the treatment of locally advanced or metastatic ER-positive/HER2-negative breast cancer, and its phase I clinical trials have started in the second half of 2019 (https://ir.arvinas.com/). Analysis of the mid-term trial showed that ARV-471 could significantly reduce ER expression level in tumor tissues, with an average of 62% and a maximum of 90%. Moreover, ARV-471 could degrade both wild-type ER and mutant ER. According to the RECIST evaluation, one patient (a total of 21 adult patients) in the ARV-471 trial had a 51% reduction in target lesion size (confirmed PR), two patients had unconfirmed PRs, and one additional patient showed stable disease, with a target lesion reduction of more than 50%. In the clinical benefit rate (CBR) evaluation, five of the 12 patients (42%) achieved CBR (defined as PRS + complete response + stable disease at 6 months).

## Conclusion and Perspectives

Different from the traditional SMIs, PROTAC is a new strategy of inducing the ubiquitination degradation of target proteins. However, it is worth noting that up to now, less than 10 of more than 600 E3 ubiquitin ligases have been used to degrade target proteins, which reminds scholars to expand their knowledge in this area.

Although the PROTAC technology has made remarkable achievements since its development in 2001, there are still some problems in the process of design and application of PROTAC. For example, the effectiveness of PROTAC depends not only on the ligands of POIs and E3 ligases but also on the length and chemical properties of the linkers connecting the ligands. In addition, the binding strength of ligands, spatial orientation, cell permeability, and other factors will have important impacts on the efficacy of PROTACs. Therefore, how these factors work together to achieve the highest efficiency is a major scientific problem to be addressed. Although the PROTACs can target protein for degradation, it cannot actively locate at the target tumor tissue and may have the off-target effects. Therefore, the safety is another challenge for PROTACs that should be taken into account. Considering the different expression of E3 ligase at different time and in different tissues, high selectivity can be achieved through tissue-specific E3 protein. After the completion of the theoretical design, several rounds of experiments are needed to optimize the structures of PROTACs and finally locate the ligands of the POIs and the E3 ligases into an appropriate spatial structure to form ternary degradation complex. It takes much time and manpower, so the application of new design strategies or technologies (e.g., CADD and AI) has a huge importance in rational design of PROTACs.

One of the biggest advantages of PROTAC technology is its great potential to target “undruggable” proteins. Because small molecule ligands can well bind to the target proteins, most of the successful PROTACs currently use SMIs as ligands to target druggable proteins. Additionally, studies by ARV-471 have clearly shown that PROTAC could produce a synergistic effect on tumor inhibition when combined with kinase inhibitors including CDK4/6 inhibitors. It suggests that combination of PROTAC either with targeted inhibitors or with chemotherapy/antibody drugs may represent a good alternative strategy for cancer therapy. It is believed that it will open up a broad road for the development of PROTAC technology and the discovery of new anticancer drugs once these problems mentioned above are solved.
